# Novel NBN-Embedded Polymers and Their Application as Fluorescent Probes in Fe^3+^ and Cr^3+^ Detection

**DOI:** 10.3390/polym14102025

**Published:** 2022-05-16

**Authors:** Tao Li, Yu-Jing Sheng, Xiao-Li Sun, Wen-Ming Wan, Yanru Liu, Qingrong Qian, Qinghua Chen

**Affiliations:** 1 Fujian Key Laboratory of Pollution Control & Resource Reuse, Engineering Research Center of Polymer Green Recycling of Ministry of Education, College of Environmental Science and Engineering, Fujian Normal University, Fuzhou 350007, China; qsz20191180@student.fjnu.edu.cn (T.L.); qrqian@fjnu.edu.cn (Q.Q.); cqhuar@fjnu.edu.cn (Q.C.); 2 State Key Laboratory of Structural Chemistry, Key Laboratory of Coal to Ethylene Glycol and Its Related Technology, Center for Excellence in Molecular Synthesis, Fujian Institute of Research on the Structure of Matter, Chinese Academy of Sciences, 155 West Yangqiao Road, Fuzhou 350002, China; shengyujing233@yeah.net; 3 School of Materials Science and Engineering, Shandong University of Science and Technology, Qingdao 266590, China; 4 College of Life Science, Fujian Normal University, Fuzhou 350007, China; yrliu@fjnu.edu.cn

**Keywords:** NBN-embedded polymers, boron-containing polymers, solvatochromic fluorescence, metal ion detection

## Abstract

The isosteric replacement of C═C by B–N units in conjugated organic systems has recently attracted tremendous interest due to its desirable optical, electronic and sensory properties. Compared with BN-, NBN- and BNB-doped polycyclic aromatic hydrocarbons, NBN-embedded polymers are poised to expand the diversity and functionality of olefin polymers, but this new class of materials remain underexplored. Herein, a series of polymers with BNB-doped π-system as a pendant group were synthesized by reversible addition-fragmentation chain transfer (RAFT) polymerization from NBN-containing vinyl monomers, which was prepared via intermolecular dehydration reaction between boronic acid and diamine moieties in one pot. Poly{2-(4-Vinylphenyl)-2,3-dihydro-1H-naphtho[1,8-de][1,3,2]diazaborinine} (P1), poly{N-(4-(1H-naphtho[1,8-de][1,3,2]diazaborinin-2(3H)-yl)phenyl)acrylamide} (P2) and poly{N-(4-(1H-benzo[d][1,3,2]diazaborol-2(3H)-yl)phenyl)acrylamide} (P3) were successfully synthesized. Their structure, photophysical properties and application in metal ion detection were investigated. Three polymers exhibit obvious solvatochromic fluorescence. As fluorescent sensors for the detection of Fe^3+^ and Cr^3+^, P1 and P2 show excellent selectivity and sensitivity. The limit of detection (LOD) achieved by Fe^3+^ is 7.30 nM, and the LOD achieved by Cr^3+^ is 14.69 nM, which indicates the great potential of these NBN-embedded polymers as metal fluorescence sensors.

## 1. Introduction

Although the rapid development of the industry has greatly enhanced the convenience of our lives, metal ion pollution in the environment is becoming increasingly serious, which is harmful to the natural environment and human health [1,2,3]. Therefore, the detection of metal ions has important practical significance today [4,5,6]. For example, iron plays an important role in many biological reaction processes, but its deficiency and excess can lead to various pathological obstacles [7,8]. Fluorescence sensors are often used in trace heavy metal ion sensing tools because of their convenient, rapid, and sensitive analysis [9,10,11,12,13]. Fluorescent probes can interact with metal ions through effects such as complexation and metal-driven chemical reactions, and at the same time, their fluorescence properties will also change, thereby achieving the effect of detecting metal ions [14,15,16]. For example, in 2019, Safarifard et al. [17]. reported the synthesis of fluorescent Zn-MOF with azine-decorated pores, an MOF which exhibited a significant quenching effect on the luminescence intensity after the introduction of Fe^3+^ ions, with a detection limit that could reach 200 nM. Feng et al. [18] reported a conjugated polymer composed of 8-methoxyquinoline and benzene, which exhibited high selectivity and sensitivity to Fe^3+^ during fluorescence quenching, with a detection limit that could reach 10 nM with strong anti-interference ability.

Boron-containing π systems have attracted considerable research attention due to their desirable optical, electronic and sensory properties [19,20,21,22,23,24,25,26]. Organic π-conjugated compounds with novel BN bonds usually have obvious optoelectronic properties/advantages due to the empty p-orbital in the boron center [27,28,29,30]. Pioneering researchers such as White, Dewar, Ashe, Liu, Schäfer, Braunschweig, and Jäkle have made considerable progress in the synthesis of BNB-doped π systems with outstanding photophysical properties [31,32,33,34,35,36,37,38,39,40]. Recently, we reported the facile and efficient synthesis of NBN-doped conjugated diazaborane and pyrene-containing NBN-doped polycyclic aromatic hydrocarbons via a catalyst-free intermolecular dehydration reaction, which has been shown to be a new class of AIEgen compounds for the sensitive detection of explosives or concentration-dependent sensory properties [41,42], but NBN-embedded polymers have rarely been studied.

Here, we synthesized three NBN-doped vinyl monomers by catalyst-free intermolecular dehydration reaction. Additionally, corresponding NBN-embedded polymers were prepared via reversible addition-fragmentation chain transfer (RAFT) polymerization, which exhibited an apparent solvatochromic fluorescence. The fluorescent NBN-embedded polymers were quenched in the presence of Fe^3+^ and Cr^3+^, thereby realizing the trace detection and the detection limit reached the nM level. These studies expanded the diversity and functionality of polymeric materials, and provided a new type of sensitive and selective fluorescent sensor for metal ion detection.

## 2. Materials and Methods

### 2.1. Materials

Azobis(isobutyronitrile) (AIBN, Aladdin-reagent, 98%, Energy Chemical, Shanghai, China) was recrystallized twice in methanol (Aladdin, Beijing, China). *S*-1-dodecyl-*S*-(*α,α*′-dimethyl-*α*″-acetic acid) trithiocarbonate (TTC) as the chain transfer agent was synthesized according to procedures detailed in the literature [43]. Tetrahydrofuran (THF, Energy Chemical, Shanghai, China) was distilled over Na/benzophenone (Energy Chemical, Shanghai, China) prior to use. 4-Vinylphenylboronic acid (VPBA, Energy Chemical, Shanghai, China), (3-Acrylamidophenyl, Energy Chemical, Shanghai, China), boronic acid (APBA, Energy Chemical, Shanghai, China), 1,8-diaminonaphthalene (Energy Chemical, Shanghai, China), 1,2-phenylenediamine (Energy Chemical, Shanghai, China) and solvents were used as received without further purification.

### 2.2. Synthesis of the Monomer

#### 2.2.1. Synthesis of 2-(4-Vinylphenyl)-2,3-dihydro-1H-naphtho[1,8-de][1,3,2]diazaborinine (M1)

A mixture of VPBA (1 g, 6.75 mmol) and 1,8-diamino-naphthalene (1.06 g, 6.75 mmol) in THF (20 mL) was allowed to react at room temperature for 12 h. Then, the residual solvent of the mixture was evaporated under reduced pressure and the crude product was subjected to silica gel column chromatography (petroleum ether:ethyl acetate (EA) = 10:1). Additionally, 1.09 g white crystals of M1 was obtained in a yield of 60%. ^1^H NMR (400 MHz, DMSO-d_6_, δ (ppm): 8.27 (s, 1H, –B–NH–), 7.91 (d, 2H, –C_6_H_4_–), 7.55 (d, 2H, –C_10_H_6_–), 7.08 (t, 2H, –C_10_H_6_–), 6.91(d, 2H, –C_6_H_4_–), 6.77 (dd, 1H, –CHCH_2_), 6.60 (d, 2H, –C_10_H_6_–), 5.96 (d, 1H, –CHCH_2_), 5.34 (d, 1H, –CHCH_2_). ^13^C NMR (400 MHz, DMSO-d_6_), δ (ppm): 142.81 (1C, –C_6_H_4_–), 139.63 (1C, –CHCH_2_), 137.14 (1C, –C_10_H_6_–), 136.41 (1C, –C_10_H_6_–), 133.47 (2C, –C_6_H_4_–), 128.12 (2C, –C_10_H_6_–), 125.86 (3C, –C_6_H_4_–), 120.15 (1C, –CHCH_2_), 116.70 (2C, –C_10_H_6_–), 115.44 (1C, –C_10_H_6_–), 106.11 (2C, –C_10_H_6_–). ^11^B NMR (600 MHz, DMSO-d_6_), δ (ppm): 30.43 (B–NH–).

#### 2.2.2. Synthesis of N-(4-(1H-naphtho[1,8-de][1,3,2]diazaborinin-2(3H)-yl)phenyl)acrylamide (M2)

APBA (1 g, 5.23 mmol) and 1,8-diamino-naphthalene (0.83 g, 5.23 mmol) were dissolved in 20 mL N,N-Dimethylformamide (DMF) and reacted for 12 h at room temperature. Then, the residual solvent of the mixture was evaporated under reduced pressure and the crude product was subjected to silica gel column chromatography (petroleum ether:EA = 4:1). Here, 0.933 g white crystals of M2 was obtained in a yield of 57%. ^1^H NMR (400 MHz, DMSO-d_6_), δ (ppm): 10.19 (s, 1H, –NH–), 8.27 (s, 1H, –B–NH–), 7.98 (s, 1H, –C_6_H_4_–), 7.78 (d, 1H, –C_6_H_4_–), 7.58 (d, 1H, –C_6_H_4_v), 7.42 (t, 1H, –C_6_H_4_–), 7.09 (t, 2H, –C_10_H_6_v), 6.93 (d, 2H, –C_10_H_6_–), 6.60 (d, 2H, –C_10_H_6_v), 6.50 (dd, 1H, –CHCH_2_), 6.28 (d, 1H, –CHCH_2_), 5.77 (d, 1H, –CHCH_2_). ^13^C NMR (400 MHz, DMSO-d_6_), δ (ppm): 163.60 (1C, –C=O), 142.80 (3C, –C_10_H_6_–), 138.71 (1C, –C_6_H_4_–), 136.39 (1C, –CHCH_2_), 132.46 (1C, –C_6_H_4_–), 128.56 (2C, –C_10_H_6_–), 128.11 (1C, –C_6_H_4_–, 1C, –CHCH_2_), 127.28 (1C, –C_6_H_4_–), 124.72 (1C, –C_6_H_4_–), 122.15 (1C, –C_6_H_4_–), 120.18 (1C, –C_10_H_6_–), 116.73 (2C, –C_10_H_6_–), 106.14 (2C, –C_10_H_6_–). ^11^B NMR (600 MHz, DMSO-d_6_), δ (ppm): 33.49 (B–NH–).

#### 2.2.3. Synthesis of N-(4-(1H-benzo[d][1,3,2]diazaborol-2(3H)-yl)phenyl)acrylamide (M3)

M3 was prepared by a similar procedure as M2, except using 1,2-phenylenediamine (0.566 g, 5.23 mmol) instead of 1,8-diaminonaphthalene. Here, 0.757 g light orange crystals was obtained in a yield of 55%. ^1^H NMR (400 MHz, DMSO-d_6_), δ (ppm):10.14 (s, 1H, –NH–), 9.09 (s, 1H, –B–NH–), 8.11 (s, 1H, –C_6_H_4_–), 7.61 (t, 2H, –C_6_H_4_–), 7.37 (t, 1H, –C_6_H_4_–), 7.06 (d, 2H, –C_6_H_4_–), 6.81 (d, 2H, –C_6_H_4_–), 6.47 (dd, 1H, –CHCH_2_), 6.26 (d, 1H, vCHCH_2_), 5.75 (d, 1H, vCHCH_2_). ^13^C NMR (400 MHz, DMSO-d_6_), δ (ppm): 163.61 (1C, –C=O), 138.94 (3C, –C_10_H_6_–),137.61 (1C, –C_6_H_4_–), 132.46 (1C, –CHCH_2_), 129.42 (1C, –C_6_H_4_–), 128.78 (1C, –C_6_H_4_–, 1C, –CHCH_2_), 127.17 (1C, –C_6_H_4_–), 125.23 (1C, –C_6_H_4_–), 121.50 (1C, –C_6_H_4_–), 118.73 (2C, –C_6_H_4_–), 111.34 (2C, –C_6_H_4_–). ^11^B NMR (600 MHz, DMSO-d_6_), δ (ppm): 27.07(B–NH–).

### 2.3. Synthesis of the Polymer

#### 2.3.1. Synthesis of P1

P1 was synthesized by RAFT polymerization in THF. Here, 0.91 g M1 (3.318 mmol), 12 mg TTC (0.033 mmol), 0.50 mg AIBN (0.003 mmol) and 2.0 mL THF in a feeding molar ratio of M1/TTC/AIBN = 1000:10:1 were added into a 10 mL Schlenk tube with a magnetic bar. After three freeze–evacuate–thaw cycles, the tube was sealed under high vacuum and immersed in an oil bath at 80 °C. After stirring for 24 h, the polymerization was stopped by immersing the Schlenk tube into ice-water and opening the tube to air. Then, the product was purified by precipitation into excessive petroleum ether, filtration and dried under vacuum overnight. Here, 0.47 g light yellow solid was obtained in a yield of 52%. The degree of polymerization (DP) of the P1 chain by ^1^H NMR analysis was determined to be 60 by comparing the integrated signal of the protons of –N*H*– (2H, –N***H***–) at 6.48 ppm and the protons in the main chain of TTC (3H, –(CH_2_)_10_C***H***_3_) at 0.82 ppm. ^1^H NMR (400 MHz, DMSO-*d_6_*), δ (ppm): 7.94 (broad, 4H, –C_6_***H***_4_–), 7.58 (broad, 2H, –C_10_***H***_6_–), 6.85 (broad, 4H, –C_10_***H***_6_–), 6.48 (broad, 2H, –N***H***–), 1.71, (broad, 1H, –C***H***CH_2_), 1.17 (broad, 2H, –CHC***H***_2_), 0.82 (broad, 3H, –(CH_2_)_10_C***H***_3_). ^11^B NMR (600 MHz, DMSO-*d_6_*), δ (ppm): 23.76 (***B***–NH–).

#### 2.3.2. Synthesis of P2

P2 and P3 were synthesized by RAFT polymerization in DMF. For P2, 0.94 g M2 (3 mmol), 11.6 mg TTC (0.03 mmol), 0.52 mg AIBN (0.003 mmol) and 2.0 mL DMF in a feeding molar ratio of M2/TTC/AIBN = 1000:10:1 were added into a 10 mL Schlenk tube with a magnetic bar. Here, 0.404 g light yellow solid of P2 was obtained in a yield of 43%. The DP of P2 chain by ^1^H NMR analysis was determined to be 48 by comparing the integrated signal of the protons of –NH– (1H, –N***H***–) at 9.82 ppm and the protons in the main chain of TTC (3H, –(CH_2_)_10_C***H***_3_) at 0.80 ppm. ^1^H NMR (400 MHz, DMSO-*d_6_*), δ (ppm): 9.82 (broad, 1H, –NH–), 7.96 (broad, 2H, –B–NH–), 7.47 (broad, 4H, –C_6_***H***_4_v), 6.93 (broad, 4H, –C_10_***H***_6_–), 6.50 (broad, 2H, –C_10_***H***_6_–), 2.01, (broad, 1H, –C***H***CH_2_), 1.17 (broad, 2H, –CHC***H***_2_), 0.80 (broad, 3H, –(CH_2_)_10_C***H***_3_). ^11^B NMR (600 MHz, DMSO-*d_6_*), δ (ppm): 24.57 (***B***–NH–).

P3 was synthesized by the same procedures. 0.371 g of P3 was obtained as an orange solid in a yield of 47%. The DP of P3 chain by ^1^H NMR analysis was determined to be 55 by comparing the integrated signal of the protons of –NH– (1H, –N***H***–) at 9.74 ppm and the protons in the main chain of TTC (3H, –(CH_2_)_10_C***H***_3_) at 0.80 ppm. ^1^H NMR (400 MHz, DMSO-*d_6_*), δ (ppm): 9.74 (broad, 1H, –N***H***–), 8.84 (broad, 2H, –B–N***H***–), 7.95 (broad, 2H, –C_6_***H***_4_–), 7.44 (broad, 2H, –C_6_***H***_4_–), 6.94 (broad, 2H, –C_6_***H***_4_–), 6.50 (broad, 2H, –C_6_***H***_4_–), 2.01, (broad, 1H, –C***H***CH_2_), 1.17 (broad, 2H, –CHC***H***_2_), 0.80 (broad, 3H, –(CH_2_)_10_C***H***_3_). ^11^B NMR (600 MHz, DMSO-*d_6_*), δ (ppm): 24.01 (***B***–NH–).

### 2.4. The Fluorescence Detection of Metal Ion

The polymers solution in THF at a concentration of 0.1 mg/mL were prepared as stock solution (P3 in DMSO). Then, 20 μL heavy metal aqueous solutions were added to 1mL stock solution, the metal ion concentration was 50 μM, and its fluorescence curves were recorded after 10 min standing (E_x_ = 365 nm). The metal ions included Na^+^, K^+^, Mg^2+^, Zn^2+^, Cu^2+^, Al^3+^, Y^3+^, La^3+^, Ln^3+^, Ce^3+^, Fe^3+^, Cr^3+^.

#### 2.4.1. The Limit of Detection (LOD)

The polymer solutions in THF at the concentration of 0.1 mg/mL were prepared as stock solution. Then, different volumes of Fe^3+^ and Cr^3+^ aqueous solutions were added to 1 ml stock solution, and its fluorescence curves were recorded after 10 min standing. The excitation wavelength was 370 nm for P1 and 375 nm for P2.

The corresponding formula was derived by fitting the data to Equation (1):

(1) $$\frac{F}{F_{0}}=1+K_{sv}C$$

where F_0_ was the initial unquenched fluorescence intensity, F was the fluorescence intensity in the presence of quencher and C was the quencher concentration.

The LOD was calculated by fitting the data into Equation (2):

(2) $$\mathrm{LOD}=\frac{3\sigma }{K_{sv}}$$

σ represents the fluorescence intensity standard deviation of blank solution, whilst K_sv_ represents the slope of the fitting curves in Figures 6B,D and 7B.

#### 2.4.2. The Time Sensitivity Test

A certain volume of metal aqueous solution containing Fe^3+^ ion / Cr^3+^ ion was added to 1mL stock solution, the metal ion concentration was fixed at 5 μM and the fluorescence curve was recorded every 15 s. The excitation wavelength was 370 nm for P1 and 375 nm for P2.

### 2.5. Characterizations 

NMR measurements were performed on a Bruker 400 MHz spectrometer (Billerica, MA, USA), whilst the ^1^H NMR spectra was obtained via Bruker 600 MHz spectrometer (Billerica, MA, USA), and the ^11^B NMR spectra was obtained by DMSO-*d_6_* using tetramethyl silane as an internal standard.

Weight-average molecular weight (*M_w_*), number-average molecular weight (*M_n_*) and the molecular weight polydispersity (PDI) of the polymers were estimated on an Agilent 1260 InfinityIIgel permeation chromatograph (GPC, Santa Clara, CA, USA) equipped with a G7110B isocratic pump and G7162A refractive index detector. Polystyrene standards were utilized, and DMF was used as the eluent at a flow rate of 1.0 mL/min at 50 °C.

Fourier transform infrared (FT-IR) data were obtained from NICOLET IS10 (Thermo Fisher Scientific, Waltham, MA, USA), which were recorded from an accumulation of 32 scans in the range of 4000~400 cm^−1^.

Fluorescence spectra were performed on RF-5301pc fluorescence spectrophotometer in a quartz cuvette with a path length of 1 cm. The slit widths of excitation and emission were 5 nm.

Thermal gravimetric analyses (TGAs) were conducted via analyzer (Q50, TA Instruments, New Castle, DE, USA) to measure the thermal stability of NBN-doped polymers. Approximately 5 mg of samples were heated from 30 to 600 °C with a heating ramp of 10 °C /min in an inert atmosphere (N_2_).

Differential scanning calorimetry (DSC) was used to determine the glass transition temperature (T_g_) of NBN-doped polymers on a TA Instruments Q20 differential scanning calorimeter (New Castle, DE, USA). The samples (5 mg by weight) were sealed in aluminum hermetic pans and subjected to a heat/cool/heat cycle over the temperature range 30~300 °C with a linear heating and cooling rate of 10 °C /min. The T_g_ of the polymer was determined from the second heating curve and analyzed using the commercially available Universal Analysis software.

### 2.6. Theoretical Calculations

All molecules were fully optimized by the density functional theory (DFT) method, where the B3LYP hybrid functional and the 6-31G* basis set were implemented in the Gaussian 09 package. Calculations used the time-dependent extension of DFT (TD-DFT) with the same function and basis set to perform vertical excitation and excited state structure optimization. The environment layer was processed using universal force field (UFF). Calculations allowed all atoms to relax in the ground state and excited state calculations.

## 3. Results and Discussion

### 3.1. Fabrication of NBN-Embedded Polymer

#### 3.1.1. Synthesis of NBN-Containing Monomer

**Scheme 1:**
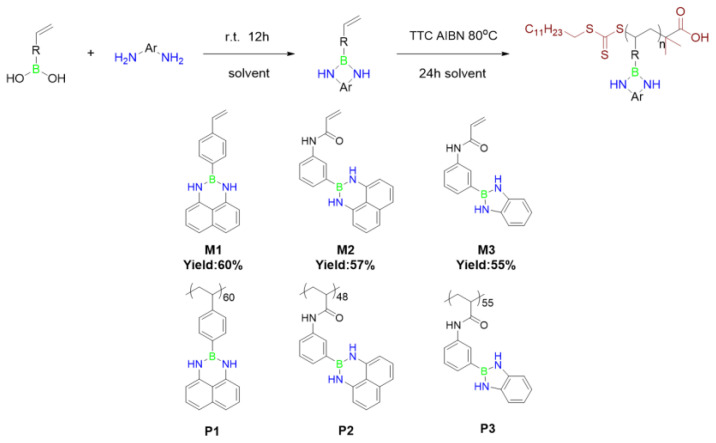
The preparation of three NBN-doped monomers and NBN-embedded polymers.

**Figure 1:**
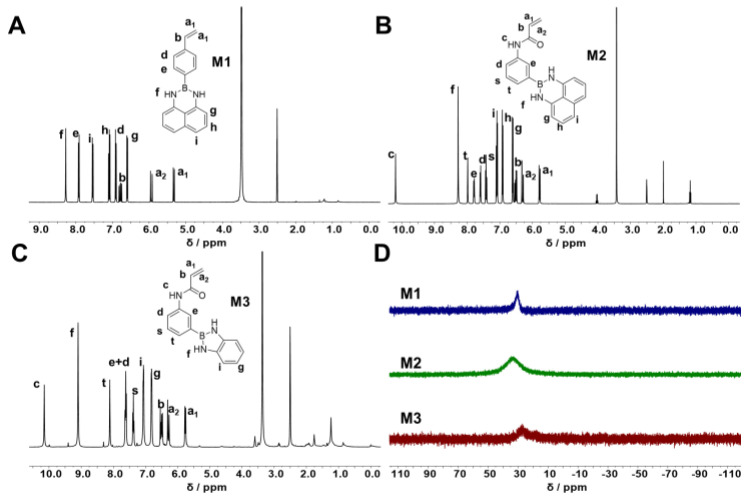
^1^H NMR (**A**–**C**) and ^11^B NMR (**D**) spectra of three NBN-doped monomers.

**Figure 2:**
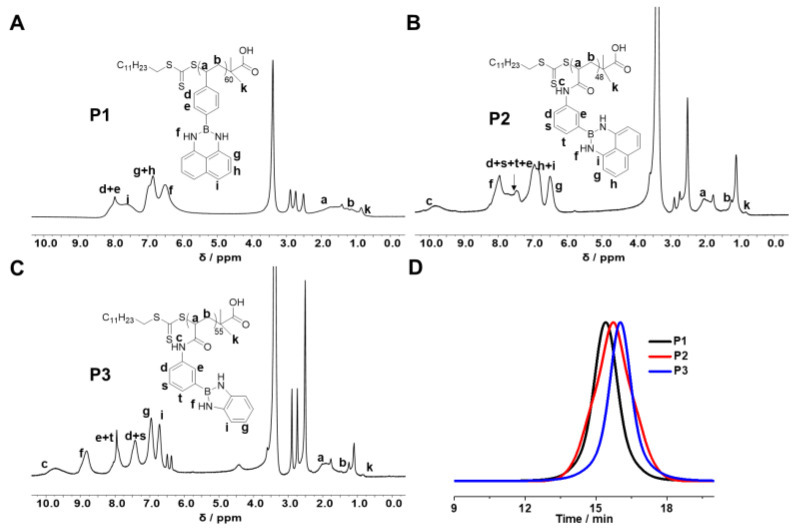
^1^H NMR spectra (**A**–**C**) and GPC curves (**D**) of NBN-embedded polymers.

NBN-doped compounds were proven to be attractive as a new type of AIEgen class for the sensitive detection of explosives or concentration-dependent sensory properties [35,36]. However, to the best of our knowledge, NBN-containing polymers have scarcely been reported. Herein, a series of polymers with a NBN-doped π system as a pendant group were first synthesized by polymerizable NBN-doped vinyl monomer and their properties were investigated. NBN-doped monomers were synthesized by a catalyst-free intermolecular dehydration reaction between boronic acid and diamine moieties at RT, as shown in Scheme 1. M1 were synthesized using VPBA with 1.8-diaminonaphthalene through one-step intermolecular dehydration, as reported in our previous work [42]. As shown in Figure 1A, the chemical structure of M1 was verified by the appearance of NBN protons at ~8.27 ppm, and by the disappearance of B(OH)_2_ protons at ~8.04 ppm of VPBA and NH_2_ protons at ~4.37 ppm of 1,2-diaminobenzene. Moreover, M2 and M3 were also successfully synthesized, as shown in Figure 2B–D.

#### 3.1.2. Synthesis of NBN-Embedded Polymers

The NBN-embedded polymers were prepared by RAFT polymerization in THF or DMF with the feeding molar ratio of monomer/TTC/AIBN = 1000:10:1, as shown in Scheme 1. For P1, the disappearance of the vinyl proton at 6.77, 5.96 and 5.34 ppm and the appearance of broad main chain signals at 1~3 ppm confirmed successful preparation, as shown in Figure 2A. The ^11^B NMR spectra also demonstrated the unique presence of the NBN group at 23.76 ppm as shown in Figure S2 (see Supplementary Materials). Further verification of the polymers was indicated by FTIR spectra in Figure S3 (see Supplementary Materials). All GPC curves are unimodal and symmetric in Figure 2D, i.e., M_n,GPC_ = 45.5k and PDI = 1.30 for P1, M_n,GPC_ = 31.3k and PDI = 1.35 for P2, M_n,GPC_ = 25.6k and PDI = 1.34 for P3. Meanwhile, as shown in Figure S4 (see Supplementary Materials), the temperature at 5% weight loss (T_d5%_) of P1 was 349.9 °C, the T_d5%_ of P2 was 200.2 °C and the T_d5%_ of P3 was 190.5 °C, which indicates that these polymers have good thermal stability. Through research on the T_g_ as shown in Figure S5 (see Supplementary Materials), the T_g_ of P1 was 227.19 °C, the T_g_ of P2 was 170.60 °C and the T_g_ of P3 was 156.80 °C. As shown in Scheme 1, three polymers have pendant NBN-doped π-systems. In P1, π-systems directly connect with main chain, while π-systems connect to the main chain through an amide group. Thus, P1 shows better thermal stability and higher T_g_.

### 3.2. Photophysical Properties

**Figure 3:**
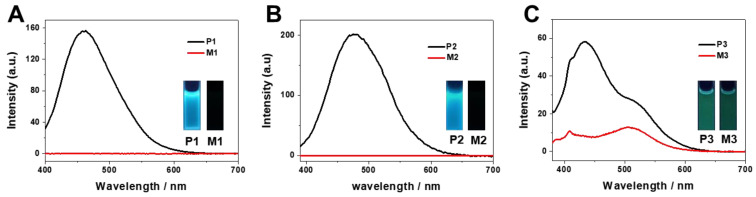
The emission spectra of M1 and P1 (**A**, E_x_ = 385 nm, in THF), M2 and P2 (**B**, E_x_ = 374 nm, in THF), M3 and P3 (**C**, E_x_ = 365 nm, in DMF) with the concentration of 0.1 mg/mL. The inset images are photos under UV light at 365 nm.

The photophysical properties of the monomers and polymers in the solution were measured as shown in Figure 3. M1 and M2 did not emitted in the solutions, including THF, DMF, EA and DMSO, while their polymers showed strong cyan fluorescence. In THF, the maximum emission wavelength for P1 is 457 nm with a quantum yield of 0.19% and P2 emits at 472 nm with a quantum yield of 0.21%. Notably, M3 has poor solubility in THF, but is well soluble in DMF, and it shows faint green emission in DMF at 505 nm with a quantum yield of 1.60%. P3 in DMF emits at 433 nm and 510 nm (shoulder peak) with a dark green color, as shown in Figure 3C, and the quantum yield achieves 7.76%. Thus, P1 and P2 with the six-membered NBN ring show similar cyan fluorescence, and P3 with the five-membered NBN ring emits green light. In our previous work, pyrene-B (o-phenylenediamine) with a five-membered NBN ring and pyrene-B (1,8-diaminonaphthalene) with a six-membered NBN ring also show differing luminescence, and the same phenomenon appeared in the luminescence of the three NBN-embedded polymers [42].

**Figure 4:**
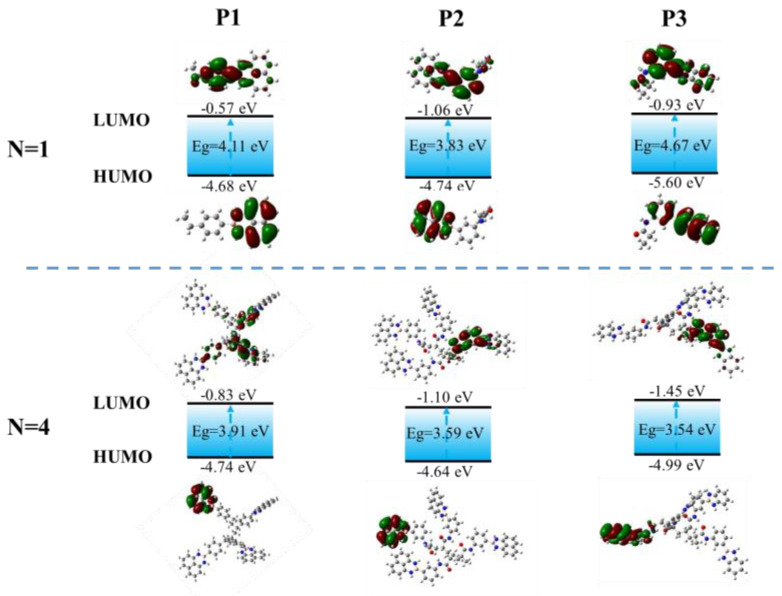
Electron cloud distributions and energy levels (eV) of NBN-embedded polymers (N = 1, 4) in the geometry-optimized S1 state calculated using the TD-DFT B3LYP/6-311G*, Gaussian 09 program.

In addition, P1 and P2 show the polymerization-induced emission (PIE) performance of non-emissive monomers, and P3 also shows enhanced emission after polymerization. Density functional theory (DFT) calculations were performed to investigate the emission mechanism of the polymer, by comparing the monomers (N = 1) and polymers (N = 4). As shown in Figure 4, all polymers show a lower band gap, resulting in the electron transition and luminescence as previously reported [44,45,46,47]. As shown in Figures S6–S8 and Table S1 (see Supplementary Materials), the band gap decreases with the N increasing for all polymers.

**Figure 5:**
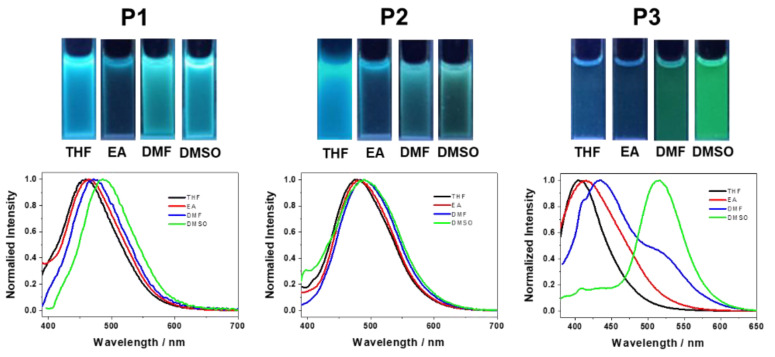
Emission spectra of the polymer solution (0.1 mg/mL) in different solvents (E_x_ = 365 nM). The illustrations are the digital photos of the polymer solutions under 365 nm lamp.

The luminescence properties of the three polymers in different solvents (THF, EA, DMF, DMSO) were investigated as shown in Figure 5, revealing the solvatochromism in their emission bands. The maximum emission of P1 was red-shifted to 460 nm in EA, 472 nm in DMF and 486 nm in DMSO with increasing solvent polarity, accompanied by an increase in the Stokes shift from 128 nm to 153 nm, as shown in Figure 5, Figure S9 and Table S2 (see Supplementary Materials). It should be noted that Stokes shifts were calculated from both absorbance and emission maxima wavelengths. The solvatochromism of P2 is similar with P1, and the emission red-shift to 492 nm in DMSO. P3 shows much obvious solvatochromism. The P3 solution shows blue fluorescence in THF and EA, dark green emission at 433 nm and 510 nm in DMF and emerald emission at 515 nm in DMSO. The quantum yield of P1, P2 and P3 in DMSO is 0.15%, 0.19% and 0.32%, respectively. As the polarity of the solvent increases, the solution of P3 shows emission with different colors, suggesting a change in the dipole moment of the molecule in transition from the ground to excited state due to the intramolecular interactions between the polar solvent and P3 [48,49,50].

### 3.3. Metal Ion Fluorescence Detection

**Figure 6:**
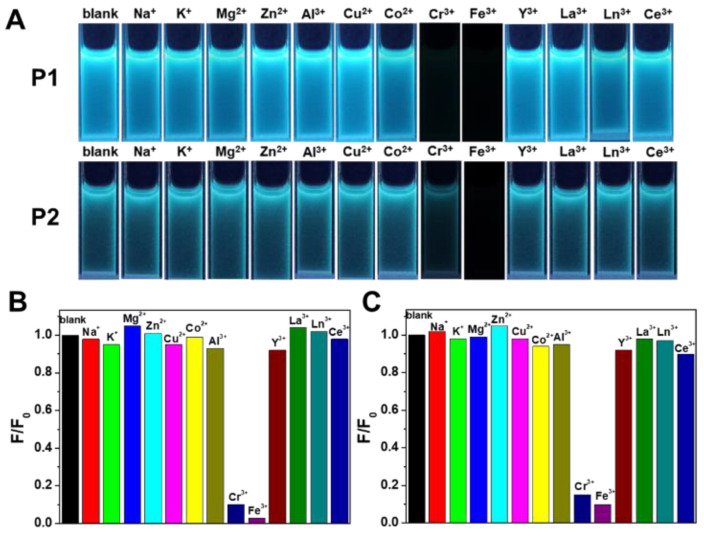
Effects of various metal ions on the fluorescence intensity of polymer solution (C_polymer_ = 0.1 mg/mL, C_metal ions_ = 50 µM). (**A**) Fluorescence photos of polymer solution versus metal ions under 365 nm UV light. The relative intensities of P1and P2 solution fluorescence (F/F_0_) versus metal ions are shown in P1 solution (**B**) and P2 solution (**C**), respectively.

For the detection of metal ions, the emission characteristics of polymers in the presence of various metal ions were investigated. Figure 6 shows the fluorescence photos of the polymer solution in THF with 50 μM metal ions and their fluorescence intensities. Metal ions, such as Na^+^, K^+^, Mg^2+^, Zn^2+^, Cu^2+^, Co^2+^, Al^3+^, Y^3+^, La^3+^, Ln^3+^ and Ce^3+^, had no obvious effect on the emission of P1 and P2 solution. However, Cr^3+^ and Fe^3+^ quenched the emission. When the Fe^3+^ or Cr^3+^ ion existed, the solution fluorescence intensity (F_0_) of the P1 solution was reduced by 97% for Fe^3+^ and 90% for Cr^3+^. For P2 solution, the cyan fluorescence was quenched by 90% in the presence of Fe^3+^ and by 85% with Cr^3+^. Meanwhile, the solution of P3 shows extensively negligible quenching in the presence of 50 μM metal ions, as shown in Figure S10 (see Supplementary Materials). It was observed that NBN-doped polymers, P1 and P2, are highly selective towards Fe^3+^ and Cr^3+^.

**Figure 7:**
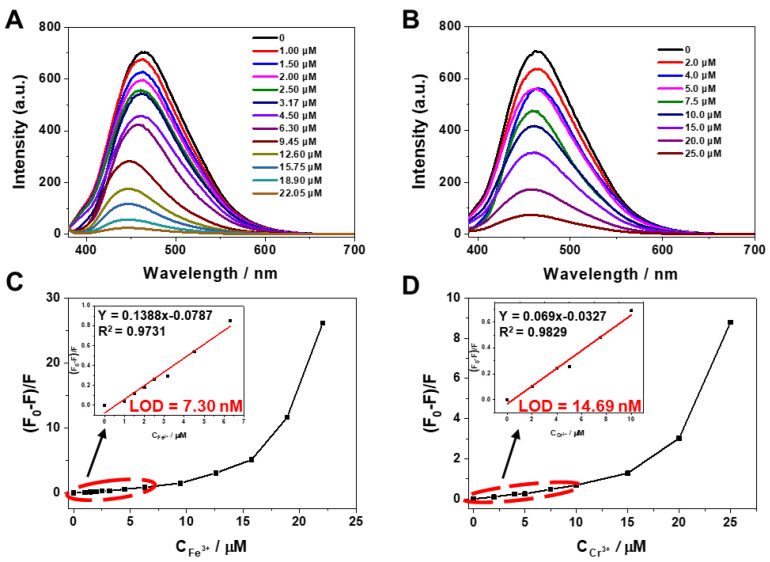
Fluorescence spectra of P1 quenched by various amounts of (**A**) Fe^3+^ (ranging from 0 to 22.05 μM) and (**B**) Cr^3+^ (ranged from 0 to 25.00 μM) in THF. Additionally, the plots of the relative intensity of fluorescence ((F_0_ − F)/F) versus the Fe^3+^ and Cr^3+^ concentrations are shown in (**C**) and (**D**), respectively.

The detection performance of P1 and P2 as a fluorescence sensor for iron and chromium was further studied. With the increase in the concentration of Fe^3+^, the fluorescence emission of the P1 solution at 457 nm gradually decreased in Figure 7A. When the Fe^3+^ concentration reached 1.0 μM, the fluorescence intensity started decreasing. When the Fe^3+^ concentration reached 22.05 μM, the fluorescence intensity decreased by 95%, and remained basically unchanged with the further increase in Fe^3+^ ion. The fluorescence calibration curve can be estimated by the Stern–Volmer (SV) equation in Figure 7C. The curve was in exponential form, indicating that both static quenching and dynamic quenching occurred during the quenching process. Notably, Fe^3+^ exhibited good linear correlation under a narrow concentration range from 1 to 6.5 μM, which indicated the static quenching at the lower concentration. In addition, the LODs based on the standard Equation (2) were calculated to be 7.30 nM. As shown in Figure S11A (see Supplementary Materials), the response time of P1 for Fe^3+^ was 45 s, which is comparable to other fluorescent materials [51]. The detection performance of P1 solution for Cr^3+^ was present in Figure 7B,D. Depending on the plot between the relative fluorescence intensity (F_0_ − F)/F and Cr^3+^ concentration, the LOD was determined to be 14.69 nM. The response time of P1 as the chemosensor for Cr^3+^ was 60 s, as shown in Figure S11B (see Supplementary Materials).

**Figure 8:**
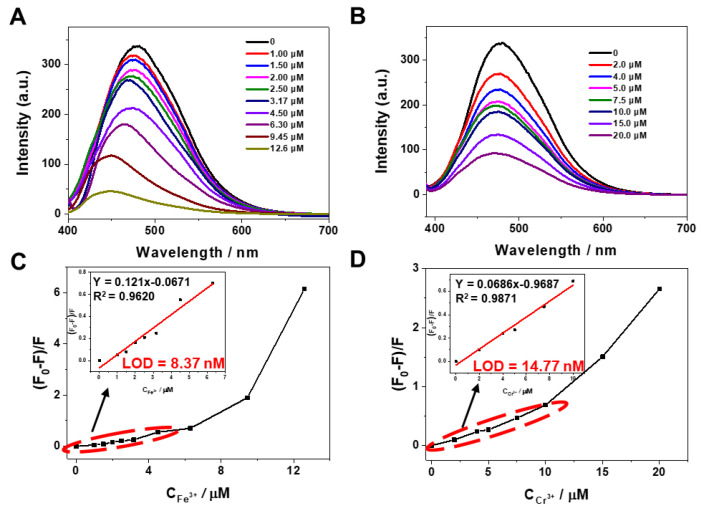
Fluorescence spectra of P2 quenched by various amounts of (**A**) Fe^3+^ (ranging from 0 to 12.6 μM) and (**B**) Cr^3+^ (ranged from 0 to 20 μM) in THF. Additionally, plots of the relative intensity of FL ((F_0_ − F)/F) versus the (**C**) Fe^3+^, and (**D**) Cr^3+^ concentrations are shown.

By the same method, the LOD of the P2 solution was calculated to be 8.37 nM for Fe^3+^ and 14.77 nM for Cr^3+^ as shown in Figure 8, and the response time was 45 s for Fe^3+^ but 90s for Cr^3+^ as shown in Figure S11C and S11D, respectively (see Supplementary Materials). Therefore, P1 and P2 showed the high sensitivity for the detection of Fe^3+^ and Cr^3+^ as the fluorescence sensor. This is probably because Fe^3+^ and Cr^3+^ coordinated with the NBN-doped six-members ring in P1 and P2, which may facilitate the charge transfer from P1/P2 to metal ions. When 15 μM EDTA was added to the polymer–ions mixture, the fluorescence intensity increased with the content of EDTA, as shown in Figure S12 (see Supplementary Materials). As a potent metal chelator, EDTA can easily combine with Fe^3+^ and Cr^3+^ to form a more stable Fe–EDTA/Cr–EDTA complex, thereby releasing the NBN-embedded polymer and their fluorescence, which confirms the quenching mechanism. The nM-level LOD demonstrated the great potential of the polymer on the metal fluorescent sensors.

**Figure 9:**
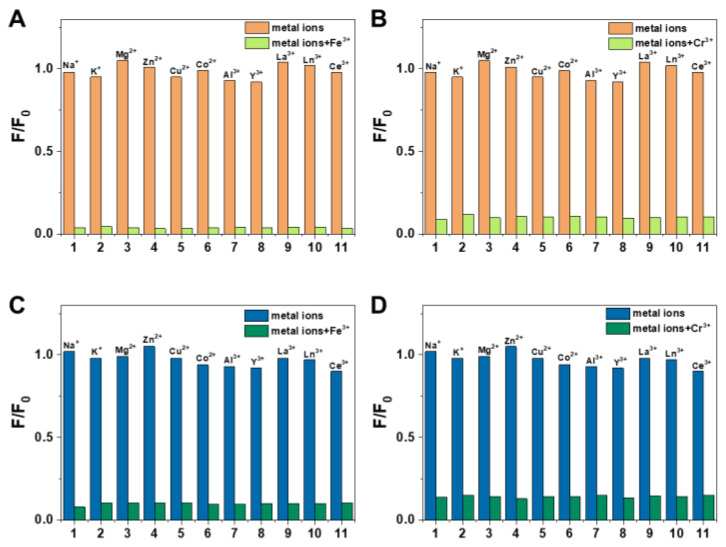
Anti-interference ability of probe P1 ((**A**) for Fe^3+^ and (**B**) for Cr^3+^) and probe P2 ((**C**) for Fe^3+^ and (**D**) for Cr^3+^) against various interferents (C_polymer_ = 0.1 mg/mL, C_ions_ = 50 µM, E_x_ = 365 nm).

The selectivity of P1 and P2 towards Fe^3+^ and Cr^3+^ was further tested as shown in Figure 9. Different competitive metal ions (50 µM, including Na^+^, K^+^, Mg^2+^, Zn^2+^, Cu^2+^, Co^2+^, Al^3+^, Y^3+^, La^3+^, Ln^3+^ and Ce^3+^) were introduced into the polymer solutions, followed by the addition of Fe^3+^ and Cr^3+^ solution. As expected, the fluorescence of polymer solutions could not be quenched by these competitive anions, but with the introduction of Fe^3+^ or Cr^3+^, the fluorescence emission was almost completely quenched. In general, the results demonstrate the high selectivity of NBN-embedded polymers towards Fe^3+^ and Cr^3+^.

## 4. Conclusions

In summary, we synthesized a series of NBN-embedded styryl and acrylamide polymers. P1, styryl polymer with a five-membered NBN ring, showed better thermal stability and higher T_g_. P1 showed blue fluorescence in the organic solvent. As acrylamide polymers with six-membered NBN ring, P2, showed blue emission in solution and P3 with five-membered NBN ring shows green emission in solution, which indicates that the fluorescence is influenced by the structure of NBN rings. All NBN-embedded polymers show polymerization-induced emission from non-emissive monomers. In addition, the polymers showed the solvatochromism dependence on the polarity of the solvent. P1 and P2 were applied as probes for the detection of metal ions. The rapid fluorescence detection of the heavy Fe^3+^ and Cr^3+^ with high selectivity and sensitivity was realized. The LOD reached 7.30 nM for Fe^3+^ and 14.69 nM for Cr^3+^ by P1, and 8.37 nM for Fe^3+^ and 14.77 nM for Cr^3+^ by P2 with excellent anti-interference ability. Therefore, as a new class of polymeric materials, NBN-embedded polymers have the advantages of being easy to synthesize and possessing admirable optical properties and sensitive detection performance, showing great prospects in polymer science.

## Data Availability

The data presented in this study are available on request from the corresponding authors.

## Supplementary Materials

The following supporting information can be downloaded at: https://www.mdpi.com/article/10.3390/polym14102025/s1, Figure S1: ^13^C NMR of NBN-doped monomers: (A) M1, (B) M2 and (C) M3; Figure S2: ^11^B NMR of NBN-embedded polymers: (A) P1, (B) P2 and (C) P3; Figure S3: FT-TR spectra of NBN-embedded polymers: (A) P1, (B) P2 and (C) P3; Figure S4: TGA curve of NBN-embedded polymers: (A) P1, (B) P2 and (C) P3; Figure S5: DSC curves of NBN-doped polymers: (A) P1, (B) P2 and (C) P3; Table S1: The E_g_ with different DP of NBN-embedded polymers; Figure S6: Electron cloud distributions and energy levels (eV) of P1 in the geometry-optimized S1 state calculated using the TD-DFT B3LYP/6-311G*, Gaussian 09 program; Figure S7: Electron cloud distributions and energy levels (eV) of P2 in the geometry-optimized S1 state calculated using the TD-DFT B3LYP/6-311G*, Gaussian 09 program; Figure S8: Electron cloud distributions and energy levels (eV) of P3 in the geometry-optimized S1 state calculated using the TD-DFT B3LYP/6-311G*, Gaussian 09 program; Table S2: Properties of three polymers in different solvents; Figure S9: The absorption spectrum of NBN-embedded polymers in different solvents; Figure S10: The fluorescence photos of P3 solution (0.1 mg/mL) with various metal ions (5.0 × 10^−5^ mol/L) in THF by 365 nm UV lamp; Figure S11: The response time of P1 for Fe^3+^ (A), Cr^3+^ (B), and P2 for Fe^3+^ (C) and Cr^3+^ (D) (C_polymer_ = 0.1 mg/mL, C_ions_ = 5 µM, E_x_ = 375 nm for P2); Figure S12: Fluorescence spectra of polymer–ions systems with EDTA addition: (A) P1– Fe^3+^, (B) P1-Cr^3+^, (C) P2-Fe^3+^ and (D) P2-Cr^3+^ (C_polymer_ = 0.05 mg/mL, C_ions_ = 10 µM, E_x_ for P1 was 370 nm and E_x_ for P2 was 375 nm); Figure S13. Fluorescence intensity evolution of probe P1 and probe P2 towards Cr^3+^ cation, Fe^3+^ cation and the mixture of Cr^3+^ and Fe^3+^ cations (C_polymer_ = 0.1 mg/mL, C_ions_ = 50 µM, E_x_ = 365 nm).
