# Supplementary material for: Novel NBN-Embedded Polymers and Their Application as Fluorescent Probes in Fe3+ and Cr3+ Detection
Source: Polymers (Basel). 2022 May 16;14(10):2025. doi: 10.3390/polym14102025 (PMC9145644; doi:10.3390/polym14102025)
Supplement: Supplementary file 1 [file polymers-14-02025-s001.zip › polymers-1711012-supplementary.pdf]

Supplementary Materials: Novel NBN-embedded Polymers and Their Application as Fluorescent Probes in Fe^{3+} and Cr^{3+} Detection

Tao Li [1,2], Yu-jing Sheng [2,3], Xiao-Li Sun [1,*], Wen-Ming Wan [2,*], Yanru Liu [4], Qingrong Qian [1] and Qinghua Chen [1]

1 Fujian Key Laboratory of Pollution Control & Resource Reuse, Engineering Research Center of Polymer Green Recycling of Ministry of Education, College of Environmental Science and Engineering, Fujian Normal University, Fuzhou 350007, China; qsz20191180@student.fjnu.edu.cn (T.L.); sunxiaoli@fjnu.edu.cn (X.-L.S.); qrqian@fjnu.edu.cn (Q.Q.); cqhuar@fjnu.edu.cn (Q.C.)

2 State Key Laboratory of Structural Chemistry, Key Laboratory of Coal to Ethylene Glycol and Its Related Technology, Center for Excellence in Molecular Synthesis, Fujian Institute of Research on the Structure of Matter, Chinese Academy of Sciences, 155 West Yangqiao Road, Fuzhou 350002, China; shengyujing233@yeah.net (Y.-J.S.); wanwenming@fjirsm.ac.cn (W.-M.W.)

3 School of Materials Science and Engineering, Shandong University of Science and Technology, Qingdao, 266590, China

4 College of Life Science, Fujian Normal University, Fuzhou 350007, China; yrliu@fjnu.edu.cn

* Correspondence: sunxiaoli@fjnu.edu.cn (X.S.); wanwenming@fjirsm.ac.cn (W.W.)

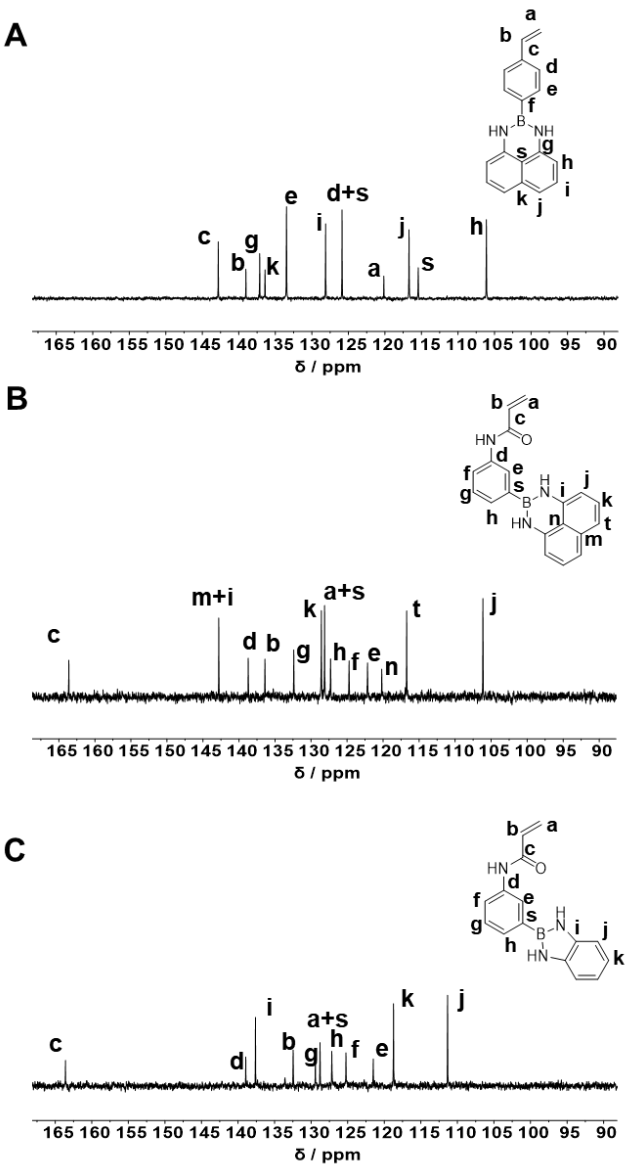

Figure S1. ^{13}C NMR of NBN-doped monomers: (**A**) M1, (**B**) M2 and (**C**) M3.

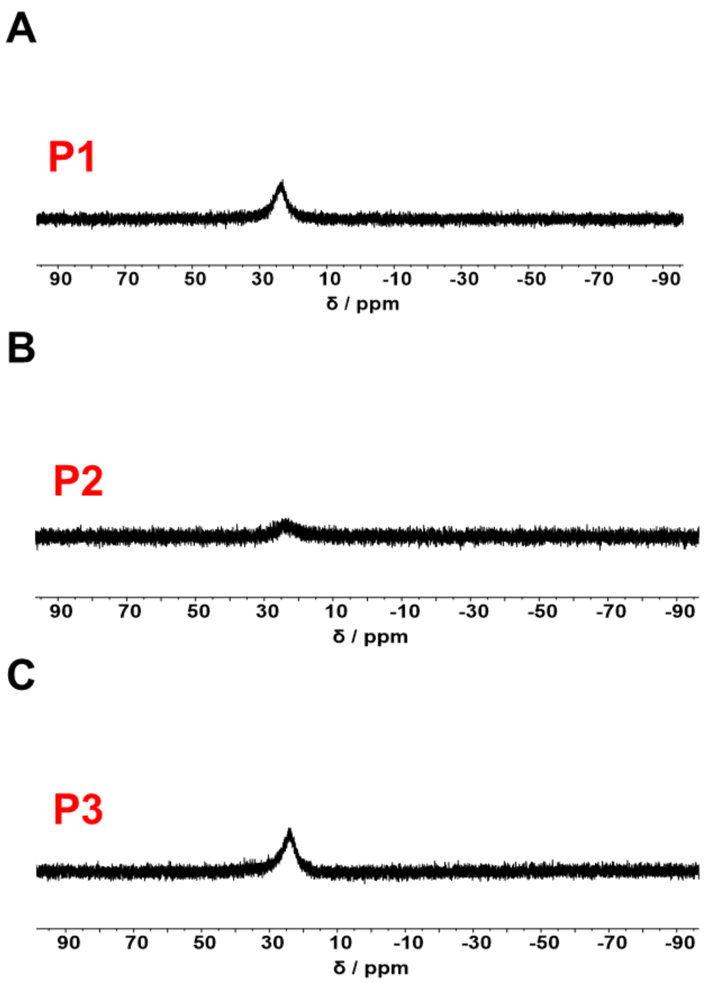

Figure S2. ^{11}B NMR of NBN-embedded polymers: (**A**) P1, (**B**) P2 and (**C**) P3.

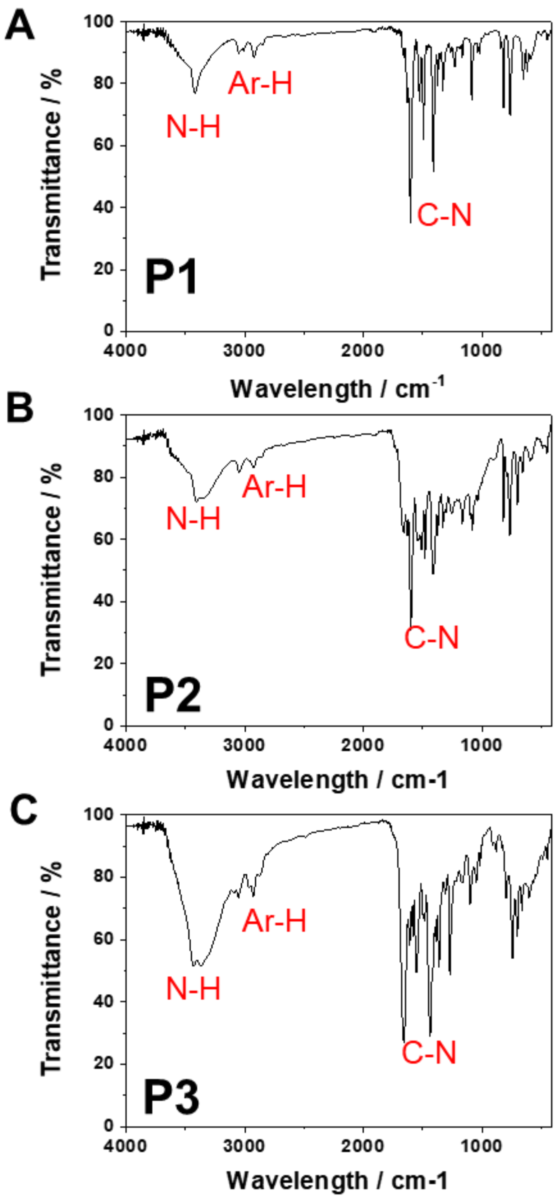

Figure S3. FT-TR spectra of NBN-embedded polymers: (**A**) P1, (**B**) P2 and (**C**) P3.

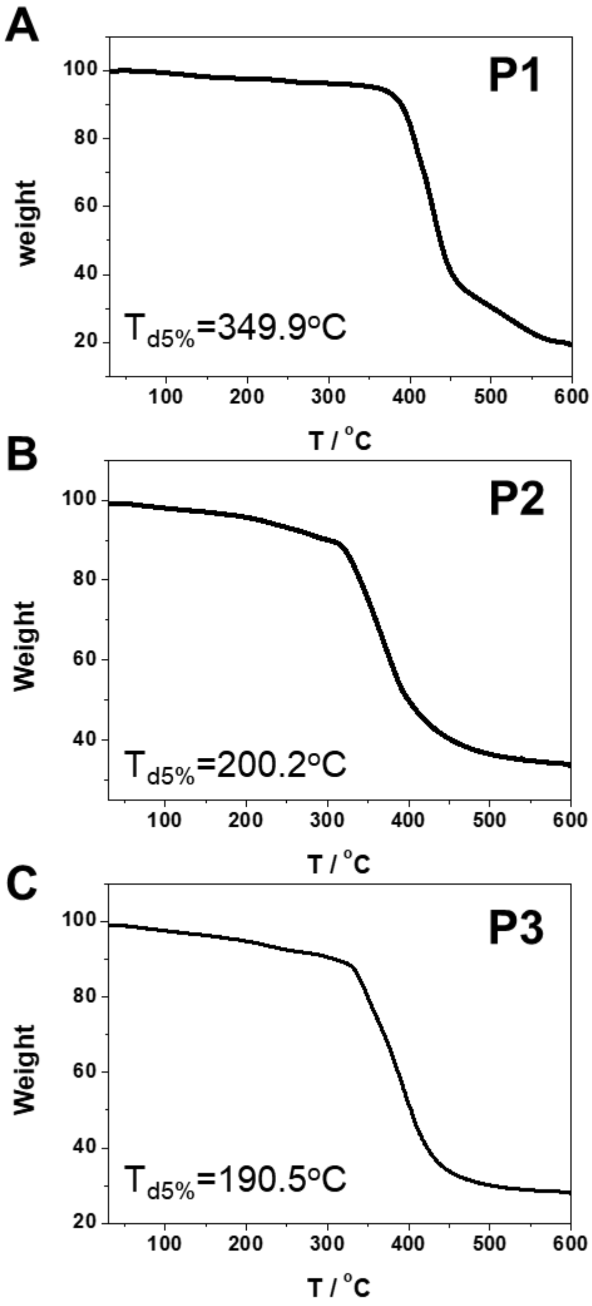

Figure S4. TGA curve of NBN-embedded polymers: (**A**) P1, (**B**) P2 and (**C**) P3.

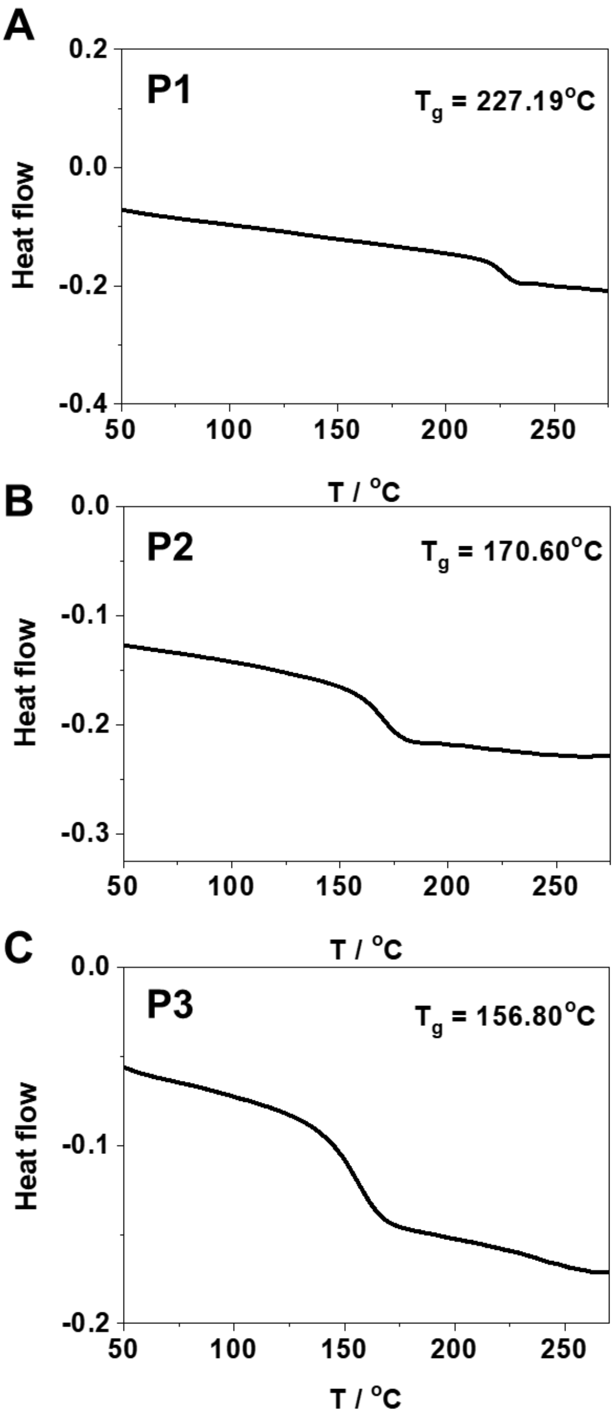

Figure S5. DSC curves of NBN-doped polymers: (**A**) P1, (**B**) P2 and (**C**) P3.

Table S1. The E_g with different DP of NBN-embedded polymers.

	Eg (eV) n=1	Eg (eV) n=2	Eg (eV) n=3	Eg (eV) n=4
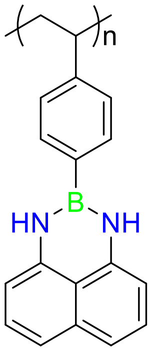	4.11	4.11	3.95	3.91
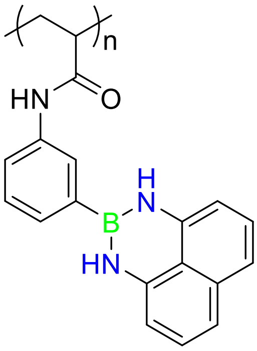	3.83	3.87	3.60	3.59
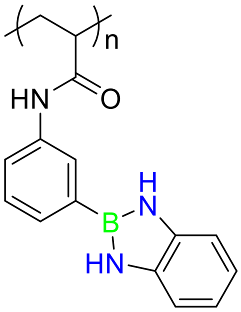	4.67	3.98	3.55	3.54

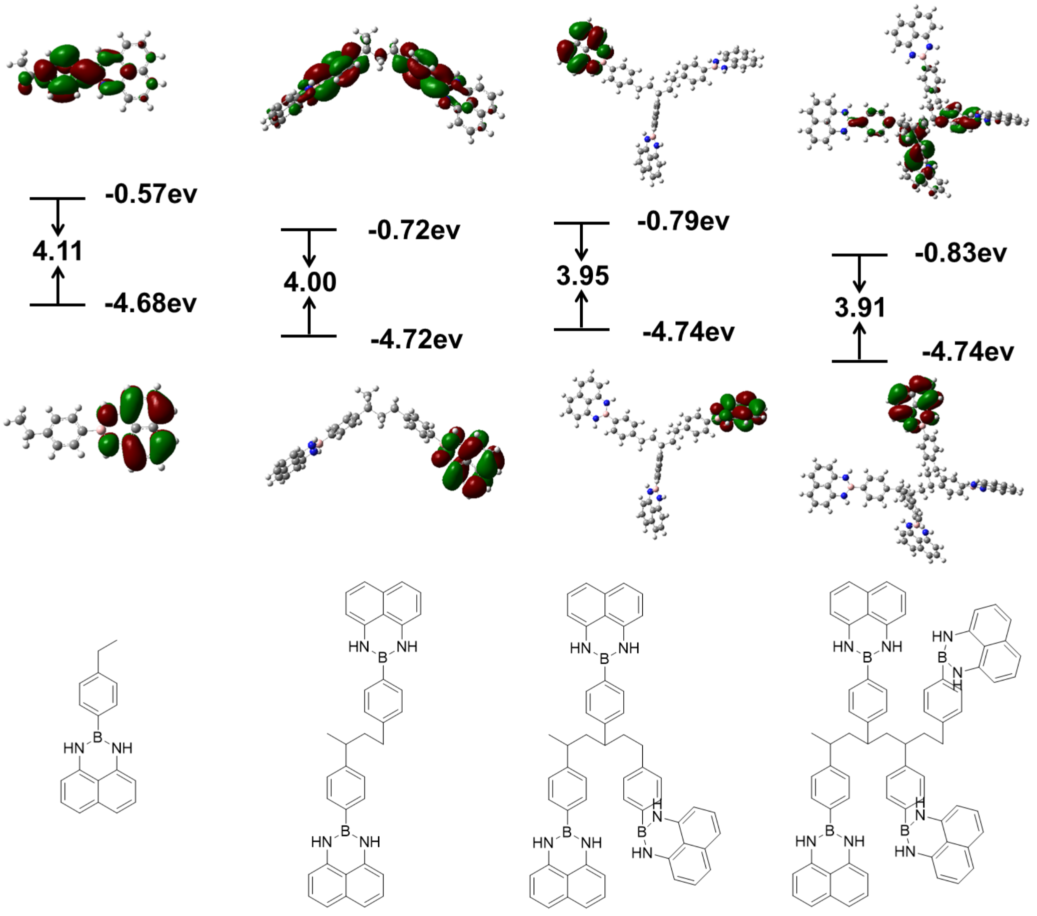

Figure S6. Electron cloud distributions and energy levels (eV) of P1 in the geometry-optimized S1 state calculated using the TD-DFT B3LYP/6-311G*, Gaussian 09 program.

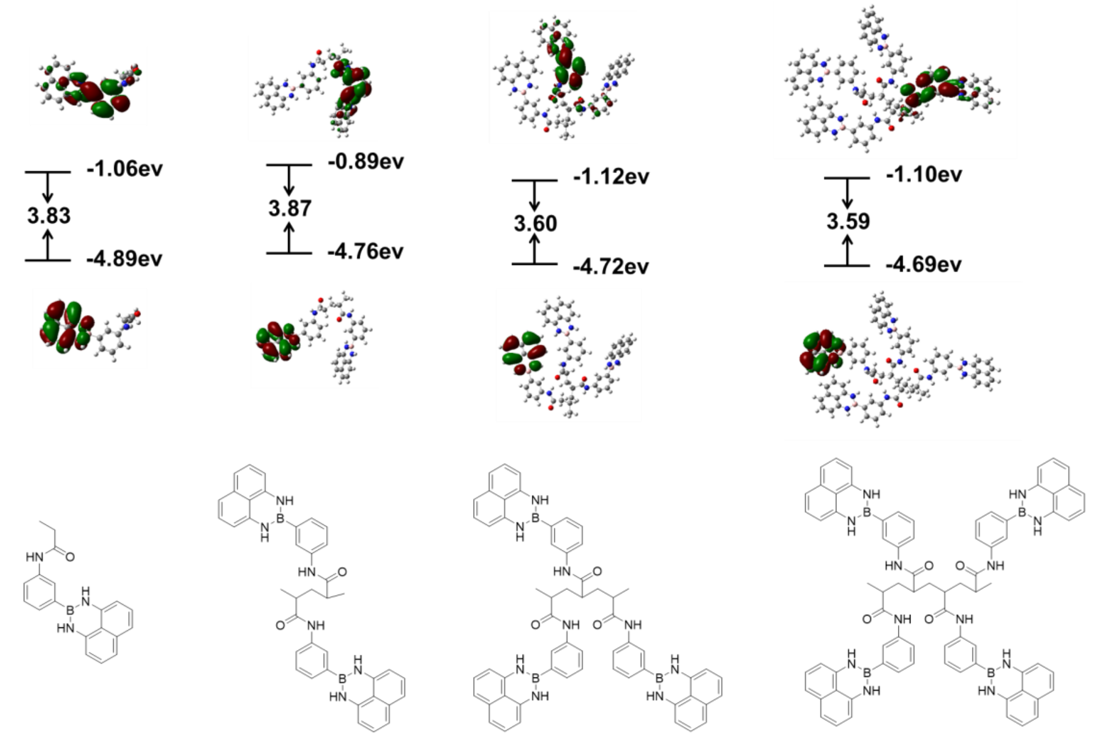

Figure S7. Electron cloud distributions and energy levels (eV) of P2 in the geometry-optimized S1 state calculated using the TD-DFT B3LYP/6-311G*, Gaussian 09 program.

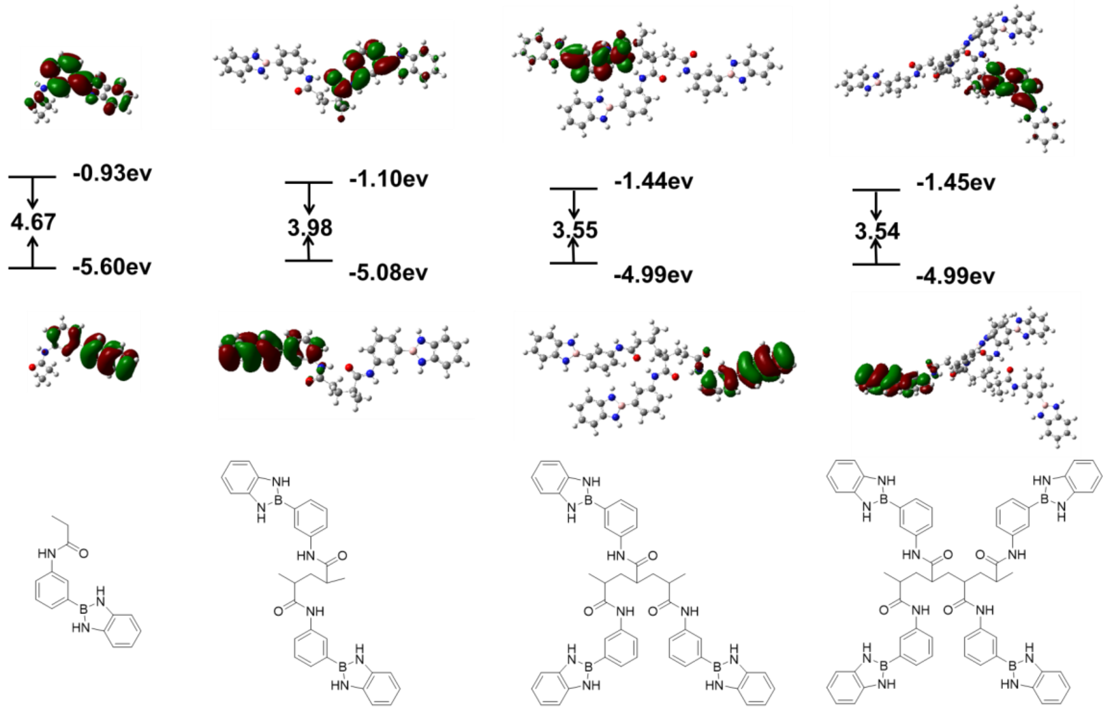

Figure S8. Electron cloud distributions and energy levels (eV) of P3 in the geometry-optimized S1 state calculated using the TD-DFT B3LYP/6-311G*, Gaussian 09 program.

Table S2. Properties of three polymers in different solvents.

Polymer	Solvent	λ_{abs} (nm)	λ_{em} (nm)	Stokes shift (nm)	Φ_f	ε (L.moL^{-1}.cm^{-1})
P1	THF	329	457	128	0.19%	35357
	EA	330	460	130		33116
	DMF	331	472	141		32467
	DMSO	333	486	153	0.15%	37337
P2	THF	333	472	139	0.21%	28846
	EA	331	481	150		30949
	DMF	333	489	156		31850
	DMSO	335	492	157	0.19%	32451
P3	THF	332	405	75		41968
	EA	332	417	85		31259
	DMF	332	433	101	7.76%	25180
	DMSO	335	515	183	0.32%	27670

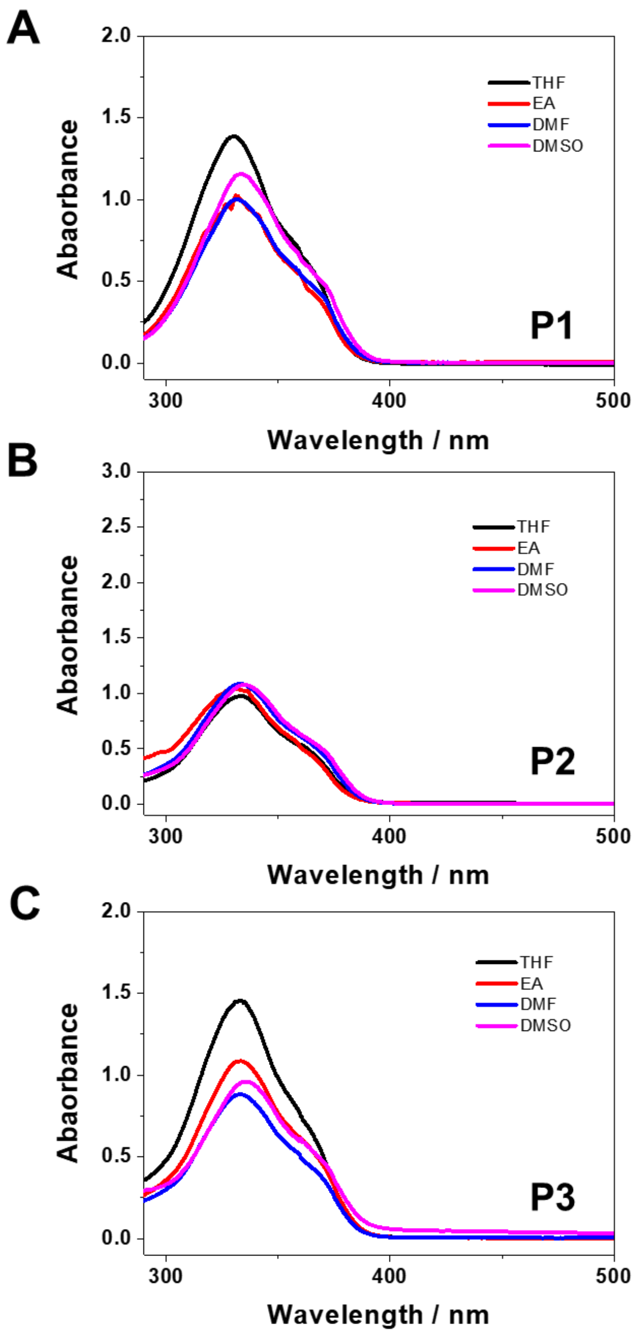

Figure S9. The absorption spectrum of NBN-embedded polymers in different solvents.

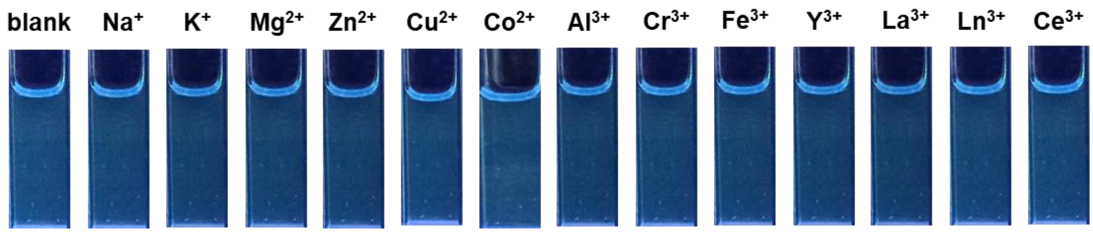

Figure S10. The fluorescence photos of P3 solution (0.1 mg/mL) with various metal ions (5.0×10^{-5} mol/L) in THF by 365 nm UV lamp.

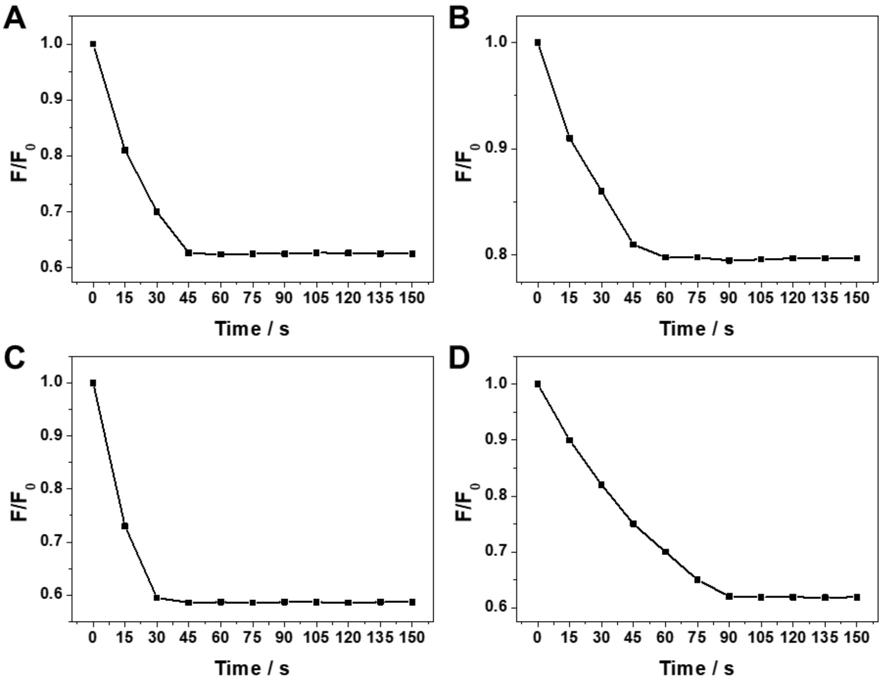

Figure S11. The response time of P1 for Fe^{3+}(**A**), Cr^{3+}(**B**) and P2 for Fe^{3+}(**C**) and Cr^{3+}(**D**) ($C_{polymer}$ = 0.1 mg/mL, C_{ions} = 5 μM, E_x = 370 nm for P1, E_x = 375 nm for P2).

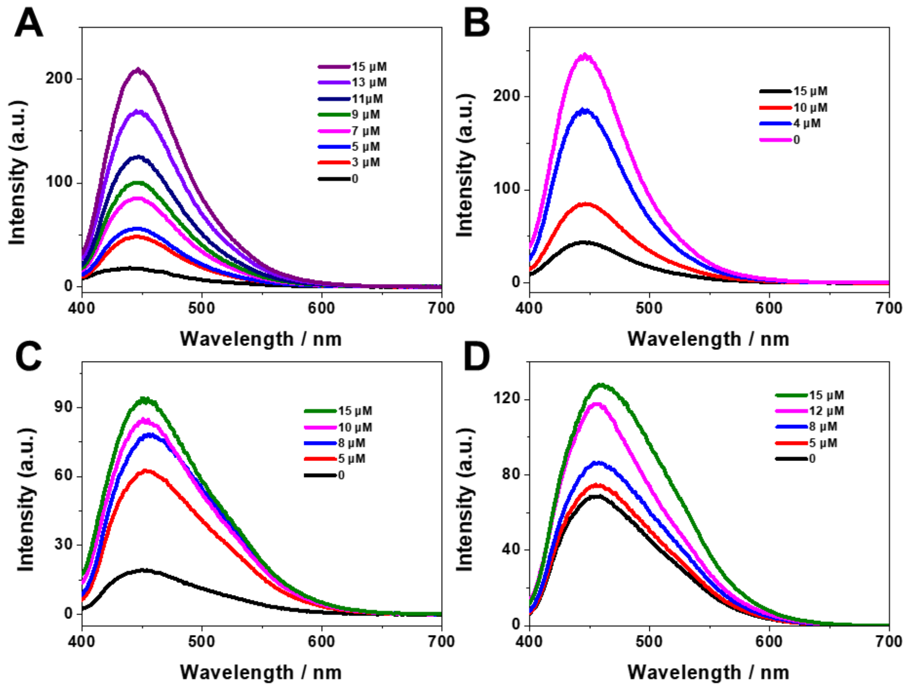

Figure S12. Fluorescence spectra of polymer–ions systems with EDTA addition: (**A**) P1–Fe^{3+}, (**B**) P1–Cr^{3+}, (**C**) P2–Fe^{3+} and (**D**) P2–Cr^{3+} ($C_{polymer}$ = 0.05 mg/mL, C_{ions} = 10 μM, E_x for P1 was 370 nm and E_x for P2 was 375 nm).

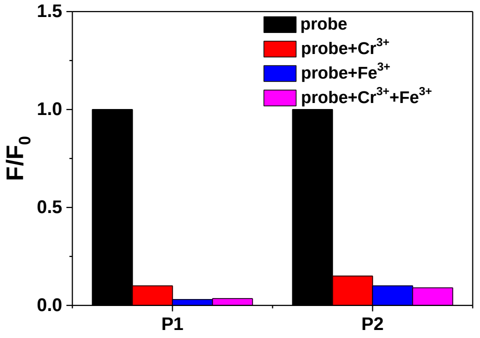

Figure S13. Fluorescence intensity evolution of probe P1 and probe P2 towards Cr^{3+} cation, Fe^{3+} cation and the mixture of Cr^{3+} and Fe^{3+} cations ($C_{polymer}$ = 0.1 mg/mL, C_{ions} = 50 μM, E_x = 365 nm).
